# Identification and Evaluation of 21 Novel Microsatellite Markers from the Autumnal Moth (*Epirrita autumnata*) (Lepidoptera: Geometridae)

**DOI:** 10.3390/ijms160922541

**Published:** 2015-09-17

**Authors:** Siv Grethe Aarnes, Ida Fløystad, Julia Schregel, Ole Petter Laksforsmo Vindstad, Jane Uhd Jepsen, Hans Geir Eiken, Rolf A. Ims, Snorre B. Hagen

**Affiliations:** 1 Norwegian Institute for Bioeconomy Research (NIBIO), Norwegian Institute for Bioeconomy Research, Svanhovd, 9925 Svanvik, Norway; E-Mails: Ida.floystad@nibio.no (I.F.); julia.schregel@nibio.no (J.S.); hansgeir.eiken@nibio.no (H.G.E.); 2 Department of Arctic and Marine Biology, University of Tromsø, 9294 Tromsø, Norway; E-Mails: ole.p.vindstad@uit.no (O.P.L.V.); rolf.ims@uit.no (R.A.I.); 3 Norwegian Institute for Nature Research (NINA), 9296 Tromsø, Norway; E-Mail: Jane.Jepsen@nina.no

**Keywords:** tri- and tetranucleotide microsatellites, multiplex PCR, Lepidoptera, population genetics

## Abstract

The autumnal moth (*Epirrita autumnata*) is a cyclically outbreaking forest Lepidoptera with circumpolar distribution and substantial impact on Northern ecosystems. We have isolated 21 microsatellites from the species to facilitate population genetic studies of population cycles, outbreaks, and crashes. First, PCR primers and PCR conditions were developed to amplify 19 trinucleotide loci and two tetranucleotide loci in six multiplex PCR approaches and then analyzed for species specificity, sensitivity and precision. Twelve of the loci showed simple tandem repeat array structures while nine loci showed imperfect repeat structures, and repeat numbers varied in our material between six and 15. The application in population genetics for all the 21 microsatellites were further validated in 48 autumnal moths sampled from Northern Norway, and allelic variation was detected in 19 loci. The detected numbers of alleles per locus ranged from two to 13, and the observed and expected heterozygosities varied from 0.04 to 0.69 and 0.04 to 0.79, respectively. Evidence for linkage disequilibrium was found for six loci as well as indication of one null allele. We find that these novel microsatellites and their multiplex-PCR assays are suitable for further research on fine- and large-scale population-genetic studies of *Epirrita autumnata*.

## 1. Introduction

The autumnal moth (*Epirrita autumnata*) is a forest pest insect with cyclic outbreak dynamics, widespread across the northern hemisphere. In the northern-boreal birch forests of Fennoscandia, outbreaks by autumnal moth and other defoliating geometrid moths, in particular winter moth (*Operopthera brumata*) but locally also the ecologically similar and recently established scarce umber moth (*Agriopis aurantiaria*), may have severe and large-scale impacts on both tree and understory layers [1,2,3,4]. This includes local or regional defoliation of mountain birch (*Betula pubescens* ssp. tortuosa) and sometimes forest death following multiyear defoliation [5]. The impact of the outbreaks may cascade through other food web compartments [4] and, occasionally, extend into neighboring tundra ecosystems [6]. These geometrid moth species are a model system in population ecology, which is partly due to the pronounced spatial population synchrony and decadal population cycles [7,8,9], but also due to the rapid outbreak range shifts shown by these three species during recent decades due to climate warming [10,11,12]. However, so far, no microsatellites have been isolated and characterized from any of these species.

Development of microsatellite DNA markers for identification and application of lepidopteran species is difficult, associated to high similarity in flanking regions between different microsatellites within the same species [13,14,15] and/or the lack of conserved flanking regions leading to unrepeatable banding patterns [16]. In addition, the sequences flanking the microsatellites have been shown to have a high incidence of single nucleotide polymorphisms and indels [17]. These properties can result in a deficit of heterozygotes due to the presence of null alleles [14]. Nevertheless, microsatellites can be used for lepidopterans with proper attention being paid to these issues [17,18,19,20]. The application of next generation sequencing of enriched genomic libraries has previously been shown to be favorable for development of short tandem repeats (STR) in Lepidoptera, as suggested in [18] and shown in several other studies [19,21,22].

Thus, we have here applied Sanger sequencing of enriched genomic libraries to identify novel *Epirrita autumnata* (*E. autumnata*) microsatellites. Furthermore, we have developed multiplex-PCR assays for 21 of those loci. We have also performed tests for species specificity, measurements of sensitivity and precision for all the 21 microsatellites, and evaluated these assays in a Northern European *E. autumnata* population. With the species specificity test we tested for cross-amplification with the two sympatric outbreak species winter moth and scarce umber moth, as a common set of microsatellites for all three species would greatly facilitate comparative population genetic studies of population cycles, outbreaks, crashes and climate driven range shifts.

## 2. Results and Discussion

Genomic screening of 192 contigs from *E. autumnata* yielded in total 90 sequences that contained an STR, and 40 STRs were selected for the development of primer sets after elimination of the dinucleotide motifs and deletion of regions where the microsatellite was too close to an end of the sequence. The 40 primer sets were tested using DNA from four moth samples to insure amplification of an appropriate amplicon. Of these, 27 were selected for detailed genotype analysis and tested on 12 field collected larvae samples as well as on 15 cultivated larvae samples. Among these 27 microsatellites, 21 showed unambiguous genotype patterns, and gave successful amplification.

**Figure 1:**
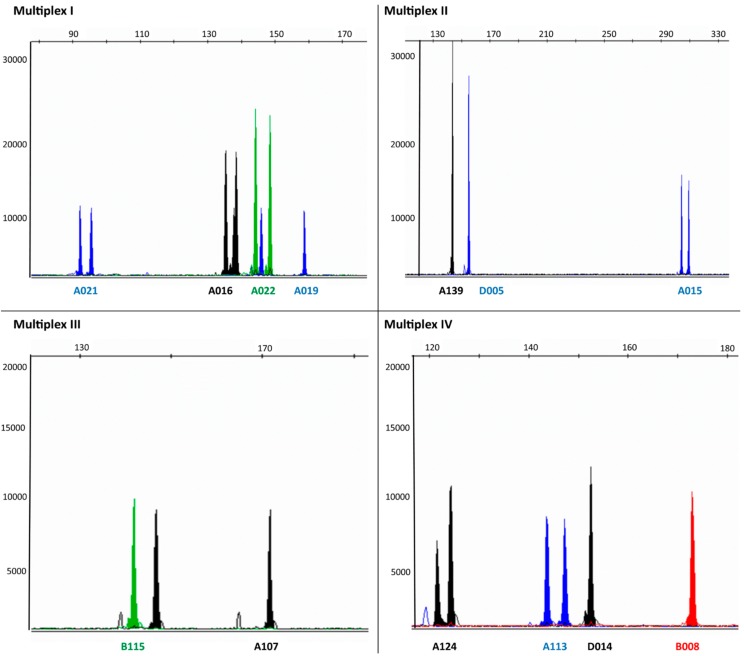

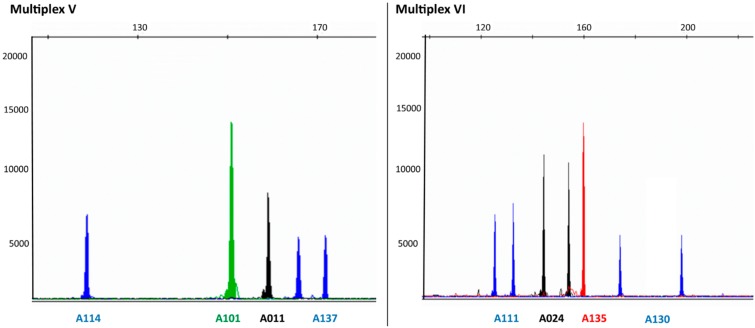
Chromatograms from capillary electrophoresis (ABI 3130xl) showing multiplex PCR reactions I–VI (see also Table 1) for the 21 microsatellites developed for *E*. *autumnata*. The peak height (Relative fluorescence units (RFU)) is indicated on the *Y*-axis, and fragment length (base-pairs) is indicated on the *X*-axis. The names of the microsatellites markers are indicated below the peaks.

The 21 microsatellites were then successfully organized into six PCR multiplex panels, making genotyping faster and cost effective, which is important if a large number of samples have to be analyzed for population genetic studies. The multiplex panels had also easily readable chromatograms with very low stutter peaks. Figure 1 shows the results obtained using these six multiplex PCRs, while primers and combination of markers used in each multiplex reaction are given in Table 1.

Amplifications for *Operophtera brumata* and *Agriopis aurantiaria* were unsuccessful, indicating species specificity of the developed primers to *E. autumnata*, and thus showing their applicability to comparative population genetic studies of these sympatric outbreak species. Development of species-specific primers for the two other species is necessary.

**Table 1:** Twenty-one microsatellite loci arranged into six multiplex PCR panels for *E. autumnata.*

Multiplex Panel	Locus	Primer Sequences (5ʹ–3ʹ) ^a^	Repeat Motif	Size Range (bp) ^b^	Primer Conc., Dye	GenBank Accession Number
I	A021	F: CCTAAGAGGGAGGCCCATGT	TGA	86–95	2 µM, FAM	KT428619
		R: CAGCTTGGTTCGTTAGCAAGG				
	A019	F: GCGTTGGCGCATCTGTAAAT	CAT	146–173	3 µM, FAM	KT428620
		R: CGCCACAGAGGTCGTCAAA				
	A022	F: CTGCGTGCTAAAACCTACGGA	CAT	141–147	1 µM, VIC	KT428621
		R: CAGCAGTGGACTTCTTCTGGC				
	A016	F: AGACCTACACCTGAGTGCATCTTAGTT	CAT	135–141	3 µM, NED	KT428622
		R: CCATCCCAGGTGTGGTGATT				
II	D005	F: CGGTGGTTGCTATGGGTGTT	CTT	142–163	2 µM, FAM	KT428623
		R: TTGCATTCTATGTGGGAGGCT				
	A015	F: AATTGTATGCCACCGCTGCT	CAT	301–325	2 µM, FAM	KT428624
		R: TCCGTCTGCCAAGTGTA				
	A139	F: ACCTGCGATTACCAATCCGA	GAT	140–149	1 µM, NED	KT428625
		R: TTCCGTGGTTCTTCTTCATCAAC				
III	B115	F: TTATAGGTGTCGGTTAAACACTTTAAAAAC	ACT	141–148	2 µM, VIC	KT428626
		R: GGTTAAGGCTGCGCTAAAGCT				
	A107	F: TAGGGCCAGCAGTGGACTTC	ATG	147–201	2 µM, NED	KT428627
		R: GTTTCTTTGGATGTCCCTTGGCCTTTA				
IV	A124	F: TGAATCGGTGCTCCAATAGGA	TAA	115–124	3 µM, NED	KT428628
		R: GTCTCTGTTGACCCCAGGGA				
	A113	F: AGACCTCGTCCAACAGTGGG	GAT	144–165	1 µM, FAM	KT428629
		R: AACATTGGACGATCTTATCGCC				
	D014	F: TCGTTTTCATCTATTATTAGTTTAGGATTCA	CAAT	140–156	2 µM, NED	KT428630
		R: TTGTTGCACGCCTTAAATGG				
	B008	F: TGCATTGTAATAGGACCTTCATATTTTT	ACAG	173–178	5 µM, PET	KT428631
		R: TTATAGGATCACTTTGTTGTCCGTCT				
V	A114	F: TGTCGAGCTCTACAAAAACTGCA	CAT	118	2 µM, FAM	KT428632
		R: AATTGGGCCTCAGGTTTCTGT				
	A101	F: GAAGCCGCGCTGTTTCTTAA	GTT	151	1 µM, VIC	KT428633
		R: GAGAGGTCGTCGAAACACCCT				
	A011	F: CTAGACCGGAGGCAAACCAA	TGA	153–159	0.5 µM, NED	KT428634
		R: CAAAATGACGGTTTGAGCGA				
	A137	F: GATCCAGGATCTGAAGCGGA	CAT	153–174	1 µM, FAM	KT428635
		R: AAGACCGTTCGTCATGGCAT				
VI	A111	F: GGCGGAGGTCTTTTCTAGCAG	ATG	122–137	2 µM, FAM	KT428636
		R: AACAAGTTTGGGTTGCAAAAGTTT				
	A130	F: AACACACTCGAGGGTCCCAA	CAT	173–227	3 µM, FAM	KT428637
		R: GTTCTAGGGCCAGCAGTGGA				
	A135	F: TCCTCCAACTCTTTCCGTGG	CAT	160–187	1 µM, PET	KT428638
		R: TTATGGGTGAGGCTTCGTCC				
	A024	F: TCGTCTGTAGATATCAACTGCTGGA	CAT	145–199	1 µM, NED	KT428639
		R: GTGGACGTAAGCAGGCTGGT				

^a^ F forward, R reverse; ^b^ Allele size range in base pairs observed in 48 individuals of *E. autumnata* (see Table 4); and Conc., Concentration.

We tested a concentration series of 20, 10, 1, 0.5, 0.2, 0.1, 0.05, 0.04, 0.03, 0.02 and 0.01 ng of template DNA in the six multiplex reactions. All markers successfully typed with signals above the lower peak height threshold of 200 RFU with template DNA in the range 20–1.0 ng. The sample material in this work had high DNA-concentration, using fresh larval head material for extraction, and the protocol were optimized to extract approximately 20 ng of DNA. This indicates a relatively low sensitivity of the assays, and for studies with other material with degraded DNA, like decades-old dried museum specimens, a new optimization is likely needed. The extraction protocol presented here should be ideal for most population genetic applications where field collected larvae or adults are used as source material.

We also tested the within-run precision using ten independent amplifications and subsequent runs of one tissue-sample of *E. autumnata* (Table 2). These results show that the standard deviations (S.D.) from allele length measurements of all the 21 loci tested were between 0.03 and 0.1 bp. The electrophoretic separation is, thus, not affected by origin of the template (Table 2).

DNA sequencing was performed on the largest and the smallest alleles in each marker found so far in the material. The sequencing revealed that all size variation observed between the two alleles selected from the same loci could be explained by variation in repeat numbers in the tandem repeat arrays (Table 3). Nineteen loci were tandem arrays of trinucleotide repeats while two had a repeat array of tetranucletide repeats (D014 and B008). Twelve of the loci showed simple tandem repeat array structures while nine loci showed complex repeat structures, often common in microsatellites in insects [23].

**Table 2:** Measurements of precision for 21 STRs from *E. autumnata*.

Locus	Allele/Genotype ^a^	Mean ^b^	S.D. (bp) ^c^
A021 allele A	92	92.24	0.07
A021 allele B	95	95.49	0.1
A019 allele A	135	135.36	0.08
A019 allele B	138	138.47	0.08
A022 allele A	145	145.92	0.08
A022 allele B	188	188.56	0.1
A016 allele A	144	144.20	0.1
A016 allele B	147	148.62	0.09
D005 allele A	148	148.43	0.07
D005 allele B	154	154.99	0.03
A015 allele A	301	301.08	0.07
A015 allele B	310	309.28	0.07
A139 allele A	143	143.53	0.07
B115 allele A	141	141.70	0.06
A107 allele A	147	146.45	0.09
A107 allele B	201	201.98	0.05
A124 allele A	121	121.44	0.06
A124 allele B	124	124.24	0.07
A113 allele A	147	147.06	0.08
D014 allele A	152	152.47	0.07
B008 allele A	181	181.21	0.06
A114 allele A	118	118.48	0.06
A101 allele A	151	150.68	0.07
A011 allele A	159	158.89	0.08
A137 allele A	165	165.68	0.09
A137 allele B	171	171.55	0.08
A111 allele A	125	125.36	0.07
A111 allele B	131	131.64	0.07
A130 allele A	173	174.16	0.07
A130 allele B	227	227.31	0.08
A135 allele A	160	159.92	0.08
A024 allele A	154	154.10	0.06
A024 allele B	172	172.09	0.09

^a^ Genotype nomenclature is based on PCR fragment sizes.; ^b^ Mean value allele sizes when measured with POP7 on ABI3730; and ^c^ SD from within-run measurement of 10 run per sample.

**Table 3:** DNA sequencing of tandem repeat structure of 21 microsatellites from *E. autumnata.*

Locus	Allele/Genotype ^a^	Number Repeats	Repeat Structure
A021 allele A	92	6 R	(TGA)_6_
A021 allele B	95	7 R	(TGA)_7_
A019 allele A	146	–	(CAT)*_n_*(AAT)(CAT)*_n_* *
A022 allele A	141 *	5 R	(CAT)_5_
A022 allele B	147 *	6 R	(CAT)_6_
A016 allele A	138	6 R	(CAT)_6_
D005 allele A	148	7 R	(CTT)_7_
D005 allele B	154	9 R	(CTT)_9_
A015 allele A	301	5 R	(CAT)_5_
A015 allele B	322	6 R	(CAT)_6_
A139 allele A	140	7 R	(GAT)_7_
A139 allele B	143	8 R	(GAT)_8_
B115 allele A	141	7 R	(ACT)_1_(TT)(ACT)_1_(ACA)(ACT)_5_
B115 allele B	144	8 R	(ACT)_1_(TT)(ACT)_1_(ACA)(ACT)_6_
A107 allele A	147	–	–
A107 allele B	201	14 R	(ATG)_6_(AGGCTG)(ATG)_3_(ACG)(ATG)_3_(CTG)(ATG)_2_
A124 allele A	121	7 R	(TAA)_7_
A124 allele B	124	8 R	(TAA)_8_
A113 allele A	147	9 R	(GAT)_9_
A113 allele B	165	15 R	(GAT)_15_
D014 allele A	140	–	(CAAT)*_n_*(CAAC)(CAT)*_n_* *
D014 allele B	142	–	–
B008 allele A	173	5 R	(ACAG)_2_(ACAT)(ACAG)_3_
B008 allele B	181	–	–
A114 allele A	118	6 R	(CAT)_6_
A101 allele A	151	8 R	(GTT)_3_(GT)(GTT)_2_(T)(GTT)_1_(GTA)(GTT)_2_
A011 allele A	159	6 R	(TGA)_3_(TGT)(TGA)_3_
A137 allele A	165	7 R	(CAT)_7_
A137 allele B	171	9R	(CAT)_9_
A111 allele A	125	10 R	(ATG)_10_
A111 allele B	134	7 R	(ATG)_7_
A130 allele A	173	6 R	(CAT)_6_
A130 allele B	227	19 R	(CAT)_7_(CAGCCT)(CAT)_6_(CAGCCTCAC)(CAT)_6_
A135 allele A	160	10 R	(CAT)_10_
A024 allele A	145	6 R	(CAT)_6_
A024 allele B	187	17 R	CTGAT(CAT)_2_(CACA)(CAT)_15_

– Sequencing not interpretable; * Structure repeat is taken from the DNA sequences done at Armalil; and ^a^ Nomenclature of alleles is based on PCR fragment size.

Allele size distribution was consistent for all STRs, except for A111, A130, B115 and D014. The irregular allele sizes observed in these STR markers are possibly caused by single base indels changing the expected sizes (see Table 3). This is also shown in other studies [17,24], and should be verified by sequencing the relevant alleles.

The six final multiplex PCR panels were applied to DNA samples from 48 *E. autumnata* from Northern Norway, and we found 98 alleles (Table 4) for all the 21 microsatellite loci, and allelic variation was detected in 19 of those, ranging from 2 to 13 (Table 4). Observed and expected heterozygosities ranged from 0.04 to 0.69 and 0.04 to 0.79, respectively.

**Table 4:** Basic statistics of 21 microsatellites loci developed for *E. autumnata* in a survey of 48 individuals from Northern Norway.

Locus	N_A_	H_O_	F_IS_	H_E_	PI	F_NULL_	HWE *p* Values ^a^
A021	3	0.438	0.1258	0.495	0.32	0.0249	0.2676
A019	3	0.553	−0.1423	0.479	0.32	−0.0496	0.9699
A022	3	0.4375	0.2654	0.5877	0.24	0.0918	0.0054 **
A016	3	0.1667	−0.0697	0.1543	0.72	−0.0111	1.0000
D005	7	0.6042	0.0347	0.6191	0.19	0.0091	0.6835
A015	5	0.4583	0.3233	0.6680	0.17	0.1322	0.0001 **
A139	3	0.229	0.1009	0.252	0.59	0.0178	0.2429
B115	5	0.354	0.2766	0.483	0.34	0.0913	0.0420 *
A107	8	0.596	0.1796	0.717	0.12	0.0571	0.0341*
A124	4	0.479	−0.0810	0.439	0.39	−0.0225	0.7396
A113	7	0.158	0.7739	0.682	0.14	0.3127	0.0000 **
D014	6	0.106	0.4148	0.179	0.68	0.0625	0.0026 *
B008	2	0.08	0.6336	0.211	0.64	0.1083	0.0194 *
A114	1	0	0	0	1.00	0	No
A101	1	0	0	0	1.00	0	No
A011	2	0.041	−0.0108	0.041	0.92	−0.0009	1.0000
A137	5	0.688	0.0061	0.645	0.16	0.0034	0.5214
A111	8	0.404	0.4519	0.726	0.12	0.1722	0.0000 **
A130	7	0.568	0.1975	0.698	0.14	0.055	0.0603
A135	2	0.255	−0.1231	0.225	0.62	−0.0246	1.0000
A024	13	0.614	0.2379	0.794	0.06	0.0833	0.0014 *

N_A_: number of different alleles; H_O_: observed heterozygosity; F_IS_: inbreeding value; H_E_: expected heterozygosity; PI: probability of identity; F_NULL_: Null-alleles estimated with the Brookfiled1 method implemented in Micro-Checker (van Oosterhout *et al*., 2006 [25]); HWE: significance of departure from Hardy–Weinberg equilibrium; * <0.05, ** <0.01; and ^a^ Based on assay of 48 individuals from each locus.

For this survey, we observed a significant linkage disequilibrium (*p* < 0.05) for nine of the 210 pairwise comparisons between loci after sequential Bonferroni correction, particularly involving loci A015, A024, A111, A130, B115 and D014 (see Table S1).

Of the nine loci that deviated significantly from HWE, three loci (A111, A113 and B008) showed large heterozygote deficiencies. These three loci also exhibited overall significant excess of homozygotes with null allele frequency of 0.172, 0.313 and 0.108, respectively, possibly indicating the presence of null allele in this population. Simulations have shown that the bias induced by null alleles is negligible at frequencies below 0.2 [26], and therefore in this population only A113 showed null allele frequency that is not negligible. Excess of homozygotes can also be due to small sample size of 48 individuals, but heterozygote deficiencies and the presence of null allele are highly common in Lepidoptera [20,27,28]. Future population studies applying these loci in a broader sampling area will help clarify both this question and whether any of the observed deviations from linkage and HW also occur consistently in other populations, which may suggest that the respective loci should be excluded from the marker set.

## 3. Materials and Methods

### 3.1. Sampling and Materials

For method development, PCR specificity and precision larval samples from a laboratory culture of *E. autumnata*, *Operophtera brumata* and *Agriopis aurantiaria* were used. The cultivated larvae originated from eggs laid by multiple females collected at Reinøya near Tromsø in Northern Norway. Thus, the cultivated larvae originated from different families within the same population. For the sensitivity test and the tests of the final set of 21 microsatellite markers (see below) we used 48 individual *E. autumnata* larvae collected at Storelva near Tromsø in Northern Norway. The 48 larvae were collected from birch trees along a linear transect with 12 individual sampling stations spaced at 200 m intervals. This was done to ensure that the larval samples were collected from a reasonably large area within the study site. Four larvae were collected from each station. Each larvae was stored individually in an Eppendorf tube and frozen at −18 °C until it was used in the DNA analysis.

### 3.2. Identification of Microsatellites Markers

A total of 40 adult moth samples of *E. autumnata* were selected and sent to Armalil Microsatellite Identification Service (www.geneticidentificationservices.com) for genome sequencing and construction of enriched genomic libraries. Here, four libraries, employing 16 capture motifs (6 TETRAs, 8 TRIs and 2 DIs), were produced using magnetic bead capture technology, and a total of 192 clones from these libraries were sequenced by Sanger sequencing using an ABI 3100 Genetic Analyzer (Applied Biosystems (ABI), Waltham, MA, USA) with a Big Dye Terminator V3.1 Cycle Sequencing Kit (ABI). Among these sequences, there were 90 microsatellite loci, and from these regions, with help of ABI Primer Express primer determining program, 40 were selected for the development of primer sets after elimination of the di-nucleotide motifs and deletion of regions where the microsatellite was too close to an end of the sequence. The 40 primer sets were tested using DNA from four moth samples to insure amplification of an appropriate amplicon. Of those tested, 27 were selected for detailed genotype analysis and tested on 16 moth samples using ABI3700 genetic analyzer.

### 3.3. DNA Extraction

DNA was extracted from head tissue of *E. autumnata* using Qiagen DNeasy Tissue kit (Qiagen, Hilden, Germany) following the manufactures’ instructions, except for the final step where we used 400 µL elution buffer to decrease the concentration of DNA. The yield of DNA was quantified using a NanoDrop 2000 (Thermo Scientific, Waltham, MA, USA).

### 3.4. Development of PCR Assays

PCR primers for the 27 loci were tested using OligoPerfect™ Designer (ABI), with the following criteria: (i) length of PCR product should be as short as possible and between 90 and 300 bp; (ii) flanking regions should not contain a mononucleotide stretch of more than five bases; (iii) annealing temperature were optimized to fall between 56 and 63 °C; and (iv) difference in temperature between forward and reverse primer should not exceed 2 °C.

Single PCRs were initially performed on 15 individuals for each of the 27 primer pairs, in a 10 µL containing 1× PCR Gold buffer (ABI), 200 µM dNTP (Eurogentec, Liège, Belgium), 1.5 mM MgCl_2_ (ABI), 0.2 µM of each primer (ABI), 1 U Amplitaq Gold DNA polymerase (ABI), 1× BSA (New England Biolabs (NEB), Ipswich, MA, USA) and 1 µL template.

DNA amplification was on an ABI 2720 for 10 min at 95 °C, 30 cycles of 30 s at 94 °C, 30 s at 56 °C, and 1 min at 72 °C, and ended with final extension for 45 min at 72 °C.

Multiplex-PCR development involved tests of different combinations of markers and primer concentration (details not shown). For the final analysis, the 21 microsatellites were split into four tetraplex (multiplex I, IV, V and VI), one triplex (multiplex II) and one diplex (multiplex III) in PCR-approach in 10 µL reaction volume using the following conditions: 5 µL 2× multiplex PCR master mix (Qiagen Multiplex kit), 0.05 µg/µL BSA (NEB) and adjusted primer set concentrations (Table 1).

PCR conditions for multiplex I–V were 10 min at 95 °C, 25 cycles of 30 s at 94 °C, 30 s at 58 °C, 1 min 72 °C and final extension for 45 min at 72 °C. PCR conditions for multiplex VI were 10 min at 95 °C, 25 cycles of 30 s at 94 °C, 30 s at 60 °C, 1 min 72 °C and final extension for 45 min at 72 °C.

PCR products (1 µL) were mixed with Genescan 500 LIZ (ABI) size standard (0.25 µL) and Hi-Di formamide (9.75 µL) following capillary electrophoresis on an ABI 3130xl Genetic Analyzer (ABI). The POP-7™ (ABI) Polymer was used as separation matrix and the sample injection time were set to 6–8 s/2 kV. PCR fragments were analyzed in GeneMapper 4.1 (ABI).

To check for possible contamination, negative controls were included for every seventh sample in all measurements in this study.

### 3.5. Testing for PCR Specificity, Sensitivity and Precision

All 21 markers were tested for cross-species amplification against DNA samples from two other species, *Operophtera brumata* (*n* = 2) and *Agriopis aurantiaria* (*n* = 2). Extraction and PCR were performed as for *E. autumnata*. Sensitivity of the six multiplex reactions in the 21 STR approach was evaluated using three samples with different amount of template DNA ranging 20–0.1 ng. Measurements of within-run precision were performed in 10 independent amplifications and subsequent runs of a single sample of *E. autumnata*.

#### 3.5.1. DNA Sequencing

The tandem repeat array and the immediate upstream and downstream sequences at each of the 21 loci were analyzed by DNA sequencing. PCR products amplified from *E. autumnata* were sequenced using the BigDye Terminator v3.1 Cycle Sequencing Kit (ABI) as recommended by the manufacturer. Forward and reverse PCR primers were used as sequencing primers in forward and reverse sequencing reactions, respectively (Table 1). Forward and reverse sequences from each sample were aligned in Sequencher 4.7 (Gene Codes Corporation, Ann Arbor, MI, USA). The allelic sequences from each locus were aligned and the sequence and size variation at each locus was determined by manual inspection. The sequence data from Armalil were used as a guidance of the suggested repeat structure.

#### 3.5.2. Analysis of Data

Analysis of Hardy–Weinberg equilibrium (HWE), expected and observed heterozygosities, population structure (F_IS_) and test for linkage disequilibrium (LD) were computed with Genetix [29]. Bonferroni corrected significance levels were applied when testing HWE and LD. Micro-Checker ver. 2.2.3 [25] was used to analyze the causes of departures from HWE: real disequilibrium, null alleles or scoring errors.

## 4. Conclusions

We find that these newly developed microsatellites and their multiplex-PCR assays are robust, fast, precise and promising to facilitate further research on fine- and large-scale population genetic studies of *E. autumnata*.

## Supplementary Materials

Supplementary materials can be found at http://www.mdpi.com/1422-0067/16/09/22541/s1.
