# Supplementary material for: Identification and Evaluation of 21 Novel Microsatellite Markers from the Autumnal Moth (Epirrita autumnata) (Lepidoptera: Geometridae)
Source: Int J Mol Sci. 2015 Sep 17;16(9):22541–54. doi: 10.3390/ijms160922541 (PMC4613323; doi:10.3390/ijms160922541)
Supplement: Supplementary file 1 [file ijms-16-22541-s001.pdf]

Supplementary Information

Table S1. Significant linkage disequilibrium ($p < 0.05$) for nine of the 210 pairwise comparisons between loci after sequential Bonferroni correction.

Linked Locus Pairs
A016–A015
A015–B08
B115–D014
B115–A024
A107–A130
D014–A111
A137–A135
A111–A024
A130–A024
